# High‐oleic acid content, nontransgenic allotetraploid cotton (*Gossypium hirsutum* L.) generated by knockout of *GhFAD2* genes with CRISPR/Cas9 system

**DOI:** 10.1111/pbi.13507

**Published:** 2020-12-29

**Authors:** Yizhen Chen, Mingchuan Fu, Hao Li, Liguo Wang, Renzhong Liu, Zhanji Liu, Xianlong Zhang, Shuangxia Jin

**Affiliations:** ^1^ Key Laboratory of Cotton Breeding and Cultivation in Huang‐Huai‐Hai Plain Ministry of Agriculture and Rural Affairs Cotton Research Center of Shandong Academy of Agricultural Sciences Jinan China; ^2^ National Key Laboratory of Crop Genetic Improvement Huazhong Agricultural University Wuhan Hubei China

**Keywords:** cotton (*Gossypium hirsutum*), genome editing, CRISPR/Cas9, high‐oleic seeds, nontransgenic

Cotton (*Gossypium hirsutum*), the most important cash crop for natural textile fibres, meanwhile, represents the fifth largest source of vegetable oil for human consumption in the world. Typically, cottonseed oil contains three major fatty acids: 26% palmitic acid, 15% oleic acid and 58% linoleic acid (Liu *et al*., 2002). The relatively high level of linoleic acid reduces oxidative stability of cottonseed oil, which can cause rancidity, a short shelf life and production of detrimental trans‐fatty acids (Shockey *et al*., 2017). Oleic acid has better oxidative stability than linoleic acid due to its monounsaturated nature, so it is considered a reliable and healthy fatty acid.

Microsomal ω‐6 fatty acid desaturase (FAD2) can introduce a carbon–carbon double bond at the Δ12 position of oleic acid to form linoleic acid (Figure 1a). Downregulation of *FAD2* via the RNA silencing method has been reported to increase oleic acid content in *Arabidopsis* and cotton (Liu *et al*., 2002). However, these transgenic lines cannot be used in any practical way due to consumer concerns about GMO and governmental regulatory issues (Shockey *et al*., 2017). Recently, the availability of versatile CRISPR/Cas genome editing techniques has allowed scientists to precisely edit the expressions of target genes without T‐DNA insertions (Wang *et al*., 2020; Zhang *et al*., 2020). Knockout of *FAD2* genes by CRISPR/Cas9 editing resulted in accumulation of about 80% oleic acid in soybean seed (Do *et al*., 2019), which represent promising example of biotechnological production of high‐oleic acid in other oilseed crops. In this study, we generated high‐oleic, nontransgenic cotton using CRISPR/Cas9 editing techniques for the first time.

**Figure 1:**
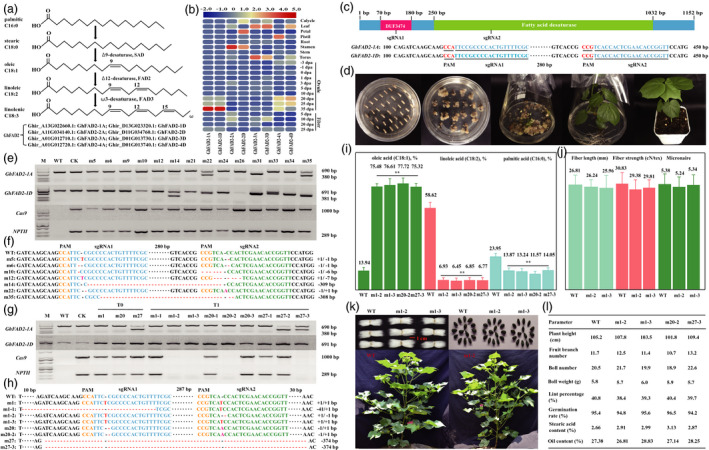
Generation of high‐oleic acid content, nontransgenic cotton plants by knockout of the *GhFAD2* genes with CRISPR/Cas9‐mediated editing system. (a) The schematic pathway of fatty acid biosynthesis in cottonseed. (b) Expression profiles of *GhFAD2* genes in 22 tissues of *G. hirsutum* TM‐1 determined by transcriptome sequencing. (c) The gene structure and target sites of *GhFAD2‐1A/D*. PAM in red letters, target sequences in blue or green letters. (d) The *Agrobacterium*‐mediated transformation and regeneration of transgenic cotton plants. (e) PCR analysis of the WT and T0 mutants. WT, the wild type Jin668. CK, Jin668 transformed by the empty vector pRGEB32‐GhU6.9‐NPT II. m, mutant. (f) Editing profile of T0 mutants based on Sanger sequencing. Deletions indicated by red dash, insertions by pink letters. (g) PCR analysis of the WT and T1 mutants. (h) Editing results of sgRNA1 and sgRNA2 in T1 mutants based on Sanger sequencing. (i) Comparison of oleic acid, linoleic acid and palmitic acid contents of WT and four nontransgenic mutants. The content of oleic acid, linoleic acid and palmitic acid was quantified with GC‐MS from 10 dry hulled cotton seeds and calculated as mg per gram dry weight. ** represents significant difference between WT and mutants at the 0.01 probability level. (j) Comparison of fibre length, strength and micronaire of the WT and two nontransgenic mutants. (k) The phenotype of the WT and CRISPR/Cas9 edited lines. (l) The major agronomic traits, seed germination and levels of stearic acid and total oil in the WT and four nontransgenic lines. Germination percentage was determined based on the results of the field trail data.

The *G. hirsutum* genome (v. HAU) encodes eight homologs (*GhFAD2*) of the *Arabidopsis FAD2* gene (Figure 1a). Among them, the GhFAD2‐1 homologs had the closest relationship with *Arabidopsis* FAD2 based on protein sequence similarity (72.30%). Transcription analysis across 22 different tissues indicated that *GhFAD2‐1A/D* expressed in the developing ovule with higher levels in the ovule at 35 days post‐anthesis (dpa), while *GhFAD2‐2A/D* highly expressed in the stamen. The expression levels of *GhFAD2‐3A/D* in the leaf and torus were relatively higher. *GhFAD2‐4A/D* expressed in all tissues of cotton with relatively higher levels in leaf, pistil and ovule at 20 dpa (Figure 1b). Taken together, these findings suggest that *GhFAD2‐1A/D* is the key gene determining the fatty acid composition of cottonseed oil.

*GhFAD2‐1A* and *GhFAD2‐1D* share the same gene structure, no introns, and contain two conserved domains, one for DUF3474 and one for fatty acid desaturase (Figure 1c). In addition, the two *GhFAD2‐1* homologs share 97.40% similarity in their amino acid sequences. We chose one target site followed by the CCA PAM motif located in the DUF3474 domain and another target site followed by the CCG PAM motif located in the fatty acid desaturase domain (Figure 1c). These sgRNAs were designed for targeting the two *GhFAD2‐1* homologs and integrated into the vector pRGEB32‐GhU6.9‐NPT II following the method described in our previous report (Wang *et al*., 2018).

We obtained 35 independent T0 plants *via Agrobacterium tumefaciens*‐mediated transformation of *G. hirsutum* genotype Jin668 (Li *et al*., 2019) (Figure 1d). Among these plants, 25 independent plants were positive transformants due to the presence of *NPTII* and *Cas9* genes (Figure 1e and g). To further investigate the editing profile of these plants, the gene regions of *GhFAD2‐1A/D* were amplified from genomic DNA of leaves by PCR using gene‐specific primers (Figure 1e and g). The results indicated that gene editing occurred at both target sites and that deletions (69.57%) were more abundant than insertions (Figure 1f). For each target site, single nucleotide insertion/deletion was predominant. A total of 86.84% of the loci were deletions of a ‘C’ in single nucleotide deletion events, while 89.29% of insertions were ‘T’ (46.43%) or ‘A’ (42.86%). As predicted, large fragment deletions of 308 and 309 bp were observed between sgRNA1 and sgRNA2 target sites (Figure 1f). Finally, 19 (76%) of the 25 T0 plants were determined to be mutants generated by the CRISPR/Cas9 system. Notably, 73.68% and 68.42% mutant T0 plants contained homozygous mutation at the sgRNA1 and sgRNA2 target sites, respectively.

Three T0 plants (m1, m20 and m27) were selected to assess the inheritance of the mutations because abundant seeds were harvested from these plants. The T1 seedlings were evaluated by PCR (Figure 1g) and Sanger sequencing (Figure 1h). The results showed all mutations induced by CRISPR/Cas9 were stably inherited to T1 generation. Interestingly, new mutations were observed in T1 plants. At the sgRNA1 target site, the m1‐1 plant showed new mutations with deletion of 41 bp, and the m1‐2 plant gained one new ‘C’ deletion at the sgRNA2 target site (Figure 1h). These complex editing patterns indicated that the Cas9 was active in T1 generation or that the T0 plants contained chimeric mutations, which is consistent with the gene‐editing profile of *BnITPK* genes in *Brassica napus* (Sashidhar *et al*., 2020). We further identified that four edited lines (m1‐2, m1‐3, m20‐2 and m27‐3) were nontransgenic due to the absence of both *NPTII* and *Cas9* genes (Figure 1g). Meanwhile, 19 potential off‐target sites were predicted using the CRISPR‐P (v. 2.0) program (Liu *et al*., 2017). The possible off‐target mutations in m1‐2 plants were further analysed *via* PCR and Sanger sequencing and no mutations were found in the potential off‐target sites, indicating the high accuracy of the CRISPR/Cas9 system.

The T1 seeds of the four nontransgenic Cas9 edited lines were subjected to fatty acid analyses. As expected, the cotton lines with knockout of *GhFAD2‐1A/D* exhibited significant increases in oleic acid at the expense of a large reduction of linoleic acid (Figure 1i). In m20‐2 seeds, oleic acid content was 77.72%, which was 5.58 (*p* < 0.01) times higher than the average level of 13.94% in wild type (WT), and the level of linoleic acid decreased concomitantly from 58.62% to 6.85% (Figure 1i). Additionally, palmitic acid contents were also significantly reduced in the four mutant lines (Figure 1i). Furthermore, to assess the impact of detected mutations on fibre quality, the fibre length, strength and Micronaire were determined and no changes were observed in the m1‐2 or m1‐3 edited lines (Figure 1j). Finally, the phenotype (Figure 1k), and levels of total oil and stearic acid in the nontransgenic seeds remained unchanged in comparison with the WT (Figure 1l). We also found that the mutant lines were not different from the WT in seed germination under normal condition, which is consistent with the germination results of high‐oleic soybean (Bachleda *et al*., 2017). These results showed that the knockout of *GhFAD2‐1A/D* had dramatically improved the quality of cottonseed oil and the high‐oleic trait has no side‐effect on major agronomic traits.

In summary, we successfully generated high‐oleic, nontransgenic mutants in allotetraploid upland cotton. This is the first report of generating high‐oleic source material in allotetraploid cotton *via* CRISPR/Cas9 editing system. These high‐oleic, nontransgenic mutants provide useful parents in breeding programs to introgress a high‐oleic trait into commercial varieties with other agronomically valuable traits.

## Conflict of interest

The authors declare no competing interests.

## Author contributions

Z.L., X.Z. and S.J. designed the study. Y.C., M.F., H.L., L.W., R.L. and Z.L. performed the experiments. Z.L. and S.J. wrote the manuscript.
